# Success Factors and Barriers in Combining Personalized Medicine and Patient Centered Care in Breast Cancer. Results from a Systematic Review and Proposal of Conceptual Framework

**DOI:** 10.3390/jpm11070654

**Published:** 2021-07-13

**Authors:** Antonio Giulio de Belvis, Rossella Pellegrino, Carolina Castagna, Alisha Morsella, Roberta Pastorino, Stefania Boccia

**Affiliations:** 1 Department of Life Sciences and Public Health, Section of Hygiene, Università Cattolica del Sacro Cuore, Largo Francesco Vito 1, 00168 Rome, Italy; antonio.debelvis@policlinicogemelli.it (A.G.d.B.); alisha.morsella@unicatt.it (A.M.); roberta.pastorino@unicatt.it (R.P.); stefania.boccia@unicatt.it (S.B.); 2 Clinical Pathways and Outcome Evaluation Unit, Fondazione Policlinico Universitario Agostino Gemelli IRCCS, Via della Pineta Sacchetti 217, 00168 Rome, Italy

**Keywords:** personalized medicine, Patient Centered Care, breast cancer management, organizational model, systematic review

## Abstract

Breast Cancer (BC) is the leading cause of death due to cancer in women. Ensuring equitable, quality-assured and effective care has increased the complexity of BC management. This systematic review reports on the state-of-the art of available literature investigating the enactment of personalized treatment and patient-centered care models in BC clinical practice, building a framework for the delivery of personalized BC care within a Patient-Centered model. Databases were searched for articles (from the inception to December 2020) reporting on Patient-Centered or Personalized Medicine BC management models, assessing success factors or limits. Out of 1885 records, 25 studies were included in our analysis. The main success factors include clearly defined roles and responsibilities within a multi-professional collaboration, appropriate training programs and adequate communication strategies and adopting a universal genomic language to improve patients’ involvement in the decision-making process. Among detected barriers, delays in the use of genetic testing were linked to the lack of public reimbursement schemes and of clear indications in timing and appropriateness. Overall, both care approaches are complementary and necessary to effectively improve BC patient management. Our framework attempts to bridge the gap in assigning a central role played by shared decision-making, still scarcely investigated in literature.

## 1. Introduction

Among today’s major public health concerns, female Breast Cancer (BC) is responsible for an estimated 2.3 million new cases every year, surpassing lung cancer as the most commonly diagnosed cancer (11.7% of all cases) and leading cause of death due to cancer in women [1]. Efforts towards guaranteeing standardized, quality-assured and effective care have led to an increasing complexity and specialization of BC management, inevitably backed by an integration of multi-specialist inputs provided collaboratively through the efforts of several healthcare practitioners. Multidisciplinary care in BC, represented by the Breast Unit model, has become the gold-standard of patient-centered BC care and has been included among the determinants for accreditation and funding of facilities, given its capacity to improve patients’ quality of life and survival rates [2,3,4].

Complementarily, advances in diagnosis and treatment have equipped clinical practice with ensembles of newly developed targeted therapies [5] and personalized medicine techniques that are gradually improving outcomes and survival, as recent research is equipping specialists with a broader understanding of the biology and heterogeneity of BC, of mechanisms behind tumour resistance and with the possibility of predicting patients’ responses to therapies, especially when dedicated clinical governance tools (i.e., molecular tumour boards) are instituted within the care pathway.

Despite such documented progress, literature seems to highlight that the application of such innovations into routine clinical practice is proceeding at a slower pace compared to the generation of such findings [6], stressing the need to concentrate efforts on their implementation into real-life settings.

The aim of this systematic review is two-fold. First, to report on the state-of-the art of available literature investigating the enactment of personalized treatment and patient-centered care models in BC clinical practice. Second, to propose a comprehensive care management framework for the delivery of personalized care to patients with BC, within a patient-centered care model.

## 2. Materials and Methods

This systematic review was conducted and reported according to the Preferred Reporting Items for Systematic Reviews and Meta-Analyses statement [7].

### 2.1. Search Strategy

The electronic databases of Web of Science, PubMed, Scopus were searched to retrieve potential eligible articles, published in English from the inception to December 2020. A Boolean search string was created using the elements of the PICO model (P, population/patient; I, intervention/indicator; C, comparator/control; and O, outcome).

Furthermore, the search on Scopus was restricted to only humans, English language, “medicine” subject area, document types—articles, reviews, editorials, letters—and publication stage—final articles. Keywords such as “breast cancer management”; “breast units”; “personalized medicine”; “patient centeredness”; “patient-centered care”; “multidisciplinary team”; “tumor board”; “molecular tumor board”; “success factors”; “limits”; “cost effectiveness” were used.

### 2.2. Inclusion/Exclusion Criteria

Articles reporting breast cancer management in terms of patient-centered care and personalized medicine approaches were considered eligible. Studies mainly discussing breast cancer clinical therapeutics, clinical epidemiological data or surgical data were excluded.

For the purpose of our study, we used the European Commission definition, according to which “Personalized medicine” is “A medical model using characterization of individuals’ phenotypes and genotypes (e.g., molecular profiling, medical imaging, lifestyle data) for tailoring the right therapeutic strategy for the right person at the right time, and/or to determine the predisposition to disease and/or to deliver timely and targeted prevention” [8].

Similarly, we used the IOM (Institute of Medicine) definition of Patient-Centered care as “care that is respectful of and responsive to individual patient preferences, needs, and values” and that ensures “that patient values guide all clinical decisions” [9].

Relying on Rotter’s 2010 Cochrane Review, the key dimensions identified for Patient Centered Care were communication [10], audit/feedback [11], informative systems [12], evidence-based application [13], multidisciplinary approach [14] and education sessions [11]. With regards to Personalized Medicine, on the other hand, the European Council’s document of 2015 identifies the molecular board [15], evidence-based genetic testing [16] and shared-clinical decision-making [17]. As such, eligible studies were those fulfilling t the definitions of patient-centered care and/or personalized medicine in terms of inclusion of the aforementioned dimensions.

In parallel, when available, relative key success factors and barriers to implementation of such dimensions were reported.

### 2.3. Study Selection

After removing duplicate records, four researchers (C.C., A.M., R.P., R.P.) independently screened by title and abstract to outline records according to the inclusion criteria aforementioned. Then, the four researchers performed a full text screening of each article to determine eligibility. Disagreements were resolved through discussion with a fifth researcher (A.G.d.B.)**.**

The reference lists of the included studies were hand-searched to locate additional studies via the snowball search method. The study selection was performed from December 2020 to February 2021.

### 2.4. Data Extraction and Synthesis

Data extraction was conducted by four independent researchers (C.C., A.M., R.P., R.P.) from the end of January to February 2021. A dedicated data extraction form on Excel was used to retrieve the following information for each eligible study:

1. Study identification (first author, title, journal, publication year);
2. Study characteristics (period, country, design);
3. Sample characteristics (stage of breast cancer, sample age, sample ethnicity);
4. Personalized Medicine and Patient centered aspects;
5. Barriers and/or success factors.

### 2.5. Quality Assessment

Three researchers (A.M., R.P., R.P.) independently conducted the methodological quality assessment, based on the different study designs. Disagreements were resolved by discussion with a fourth researcher (C.C.)**.** The standardised critical appraisal tool (1) Scale for the Assessment of Narrative Review Article SANRA [18] was used to assess narrative reviews; (2) Critical Appraisal Skills Programme, CASP [19] for qualitative research; (3) JBI Critical Appraisal Checklist for cohort studies [20]; (4) JBI Critical Appraisal Checklist for text and opinion [21]; (5) The Newcastle-Ottawa Scale NOS for cross-sectional studies [22]; (6) JBI Critical Appraisal Checklist for systematic review and research syntheses [23]. To summarise the overall evidence quality, we decided to cluster the records retrieved into three categories, based on the number of quality criteria met: group 1, studies satisfied at least 75% of the quality criteria; group 2, studies encountering between 55% and 74% of the quality criteria; and group 3, studies met less than 55% of the quality criteria.

## 3. Results

A total of 1885 records were collected from all the databases searched. After removing duplicates, the remaining 1806 articles were screened by title and abstract. The full texts of the 83 retained papers were screened. Of these, 25 met the eligibility criteria and were included in the systematic review and in the qualitative analysis.

**Figure 1:**
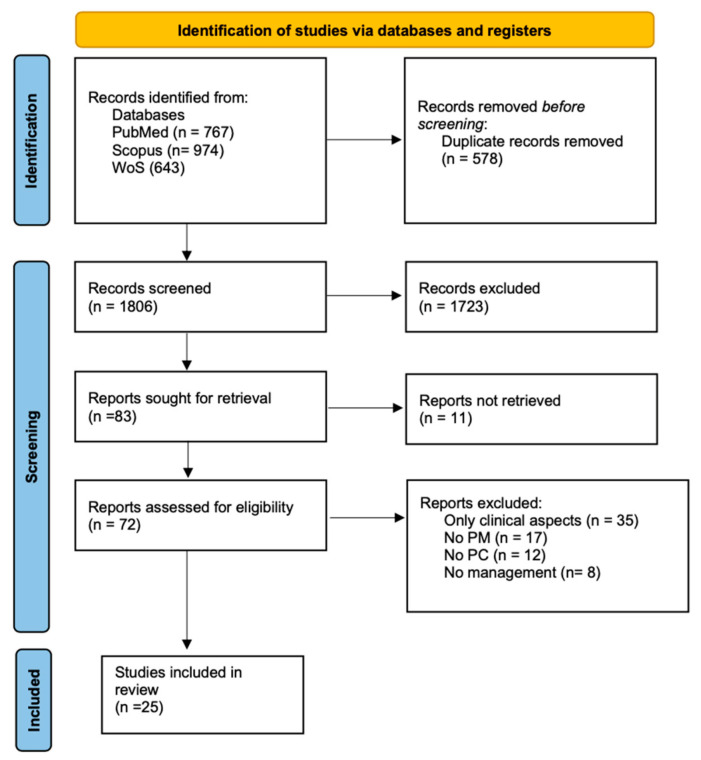
PRISMA Flowchart of the literature review and selection process for breast cancer management in terms of patient-centered care and personalized medicine approaches.

The process of study screening and selection is reported in Figure 1.

### 3.1. Results of Quality Assessment

The majority of the included articles were narrative reviews [24,25,26,27,28,29,30,31,32] (36%) and qualitative researches [33,34,35,36,37,38,39] (28%). As reported in Table 1, six [24,25,27,34,40,41] out of 25 articles (24%) were classified in group 1, since they satisfied at least 75% of the quality criteria, while the majority—11 [26,31,33,35,37,38,39,42,43,44,45] out of 25 articles (44%)—were allocated in group 2, meeting between 55% and 74% of the quality criteria. Lastly, 8 [28,29,30,32,36,46,47,48] out of 25 studies (32%) were inserted in group 3, satisfying less than 55% of the quality criteria.

### 3.2. Outcome Categories

Below, we report findings relative to the key dimensions of Patient Centered Care and Personalized Medicine, alongside relative key success factors and barriers as shown in Table 2.

#### 3.2.1. Patient-Centered Care Key Dimensions

The patient-centered care category included five [27,36,37,42,44] out of twenty-five articles (20%). Findings relative to the key dimensions were:

- Communication: the description and analysis of communicative skills of clinicians [36] (20%);
- Audit and feedback: the measurement of quality indicators and compliance with the standards proposed by the European Society on Breast Cancer Specialists (EUSOMA) [27,42] (40%);
- Informative systems: their utility and effectiveness for management of cancer-related data [37] (20%);
- Evidence-based application (guidelines, critical pathway, and procedures): the improvement of patients outcome required necessarily the adherence to the guidelines [42,44] (40%).

#### 3.2.2. Personalized Medicine Key Dimensions

Eleven [24,28,30,32,33,34,35,41,43,45,46] out of twenty-five articles (44%) provided information on personalized medicine for the treatment of breast cancer. Results about key dimensions were:

- Molecular board (compositions and conditions for efficacy): the role, responsibilities and tasks of all members, in particular pathologists and surgeons [24,28,32,33,35] (45%);
- Evidence-based genetic testing: the benefits and clinical validity of -omics technologies, focusing on *BRCA* and Oncotype DX [24,30,32,34,35,41,43,45,46] (82%);
- Shared-clinical decision-making: a positive impact on the selection of targeted therapy and improvements in chemotherapy, endocrine, and chemo-endocrine therapy decisions [24,30,32,34,35,41,43,45,46] (82%).

Only three [24,32,35] out of eleven articles (27%) provided information on all three dimensions, indicating that the combination of these would be necessary and essential to ensure a more accurate and efficacy targeted treatment.

#### 3.2.3. Key Dimensions for Applying Personalized Medicine and Patient-Centered Care

Nine [25,26,29,31,38,39,40,47,48] out of twenty-five articles (36%) reported information on both categories (PM and PC).

In particular, two [40,47] out of nine articles (22%) referred to the molecular board; eight [25,26,29,31,38,39,47,48] (89%) referred to evidence-based genetic testing.

Information on shared-clinical decision-making was reported by six [26,29,31,38,47,48] (66%): in particular, only one [31] described and analyzed several web supports to include the patient in the decision-making process.

Four [29,38,39,40] (44%) referred to communication, focusing on a greater sharing of information during genetic counselling.

One [48] (11%) referred to audit/feedback and two [26,39] (22%) to informative systems. Both agreed on the introduction of scientific tools, such as electronic medical records (EMR), to combine clinical and genomic data.

Evidence-based application was described by only one study [48] (11%), with the implementation of an integrated oncology clinical pathway model (IOCP).

Seven [25,26,29,31,39,40,47] (78%) referred to a multidisciplinary approach: most of them agreed on the need for education and training programs about genetic testing for all members of the multidisciplinary team.

### 3.3. Success Factors and Barriers in the Management of Breast Cancer Pathway

#### 3.3.1. Success Factors

Analysis of the included articles revealed the following key success factors of the enactment of personalized treatment and patient-centered care models in BC clinical practice, which can be summarized as follows: multiprofessional/multispecialist collaboration; education and training on genetic testing; utilization of personalized medicine prevention techniques; clear communication between clinicians and patients; data derived benchmarking for quality improvement.

More specifically, appropriately training and assembling multi-professional and multi-specialist teams is described as an organizational strength for the treatment of BC [26,28,31,32,33,47]. In breast oncology practice, in fact, the team must necessarily include qualified specialists to bridge the gap between clinical knowledge and genetic potential, especially able to collect and evaluate genomic profiling data [31,33]. The importance of refining clinicians’ training on genetic testing and communication strategies is also linked to clinician-to-patient communication [36,38]: a communicative approach focused on patients and their background consent a better involvement of them in the decision-making process [43]. In addition, measuring and benchmarking the performance of clinical pathways through quality indicators allow us to identify, on one side, weak areas that require improvement and, on the other, those which adhered to clinical guidelines [42,44,48].

Lastly, another key success factor is found in the appropriateness of the genetic test selection process, which must take into account clinical utility and validity, analytical validity, as well as ethical, legal, and social implications [25].

#### 3.3.2. Barriers

Our records allowed us to identify some general barriers that should be improved and overcome in the future, such as the lack of a universal language among health professionals, the uncertain composition of multidisciplinary teams and delays in the adoption of new technologies in clinical practice.

The need for proper training and education for geneticists and clinicians [24,25,39] is not supported by the adoption of a universal genomics vocabulary [41]. Additionally, the unclear definition of the scope of the genetic counseling—or an insufficiently involved geneticist—represents a barrier to proper and adequate communication with the patient [33,38]. From here, the need to define the role of genetic counseling through internal protocols, both before and after the execution of the tests, also serves as a barrier [40].

Moreover, there seems to be lack of clarity on the allocation of tasks and responsibilities among all members of the multidisciplinary team. Especially between oncologists and geneticists, the administration, interpretation and use of genetic tests and -omics technologies are fragmented [35], further confirming the importance of introducing new professional profiles trained and responsible for data collection and analysis, such as data manager [37].

The delay in the adoption of -omic technologies in clinical practice is associated to the lack of clarity in their appropriate timing and use [39,41,44]. Furthermore, the insufficient use of genetic testing is linked to the absence of public reimbursement schemes which disincentivizes their utilization [34,46].

## 4. Discussion

Authors should discuss the results and how they can be interpreted from the perspective of previous studies and of the working hypotheses. The findings and their implications should be discussed in the broadest context possible. Future research directions may also be highlighted.

We systematically reported on the state-of-the-art of available literature investigating the enactment of personalized treatment and patient-centered care models in BC clinical practice. We also aimed at highlighting the key success factors of such a shift, alongside relative barriers to implementation. The main success factors found in our included articles are the clear definition of roles and responsibilities within a multi-professional collaboration, backed by appropriate training and education programs. Last but not least, there is the need for an adequate communication strategy which includes the adoption of a universal genomic language and the active involvement of the patient in the decision-making process. Overall, it appears that -omics technology cannot rely on a solid organizational structure, neither at the micro level of single facilities or at the macro level of the health system. Compared to El-Alti et al. 2019 [49], which questions whether the relationship between personalized medicine and patient-centered care is a complementary or mutually exclusive one, our study takes a step further and answers the question by supporting the thesis by which these models are indeed synergically complementary.

In fact, PM alone is not sufficient to select the best treatment choice as it focuses mainly on somatic, biological and genetic features of the patient. It is here that the role played by Patient Centered Care ensures the active engagement of the patient by bringing his personal needs and preferences into the decision-making process. Nardini et al. 2021 [50] describe a new healthcare configuration that includes personalized, predictive, preventive, participatory and person-centered care approaches, to be applied to all areas of medicine. Agreeing with such findings, this review recognizes how both models together are needed to ensure a positive and effective change in the path of patient care and attempts to tailor such a transition to the context of breast cancer care.

With the next step being the delivery of personalized care, it becomes central to find solutions to the array of organizational challenges that come with it to allow, on one side, the high degree of specialization, scientific-technical advances and multidisciplinary and multiprofessional coordination, and, on the other, patient participation and the ability to respond to multiple care needs [51].

**Figure 2:**
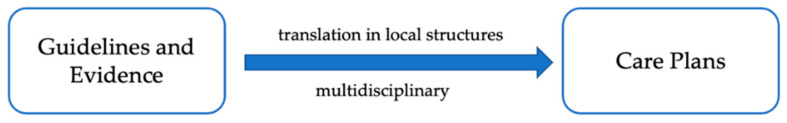
The “classic” clinical pathway on breast cancer.

Internationally, clinical pathways have been recognized as a suitable tool to drive care towards patient-centeredness, and result from a translation of Evidence Based Medicine (EBM) principles into localized contexts (Figure 2), as a strategy to reduce unnecessary clinical variability, costs, fragmentation of care improving outcomes and quality of care [52]. However, it has been argued that committing to one or a few best recommendations and standards may slow down the pace of change in the landscape of oncological care and prevent providers and professionals from being able to deliver individualized treatments that synergize with patient preferences [53].

It is in the recognition of such limitations that it becomes necessary to bring personalized medicine into clinical practice by rendering it an integral part of clinical pathways. Their nature, in fact, allows for merging the ultimate therapeutic advancements and best current evidence with individual patient characteristics and preferences, while still attenuating unnecessary clinical variability.

Typically, patient preferences are left out in the construction process of the “classic Clinical Pathway model” as they are not considered among principal factors. However, when therapeutic options may lead to different results depending on patients’ preferences, patients’ participation in the decision-making process is a keystone of high-quality cancer care. Shared Decision-Making (SDM) can be defined as “an approach where physicians and patients share the best available evidence when faced with the task of making treatment decisions, and where patients are supported to consider options, to achieve informed preferences [54], with positive effects on patient satisfaction cost effectiveness and the number of malpractice lawsuits” [55,56,57] (Maes-Carballo et al., 2020) [58].

**Figure 3:**
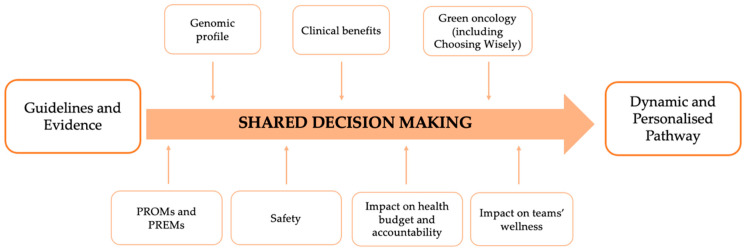
The personalized pathway on breast cancer.

In light of the above, the BC Clinical Pathway constitutes an ideal context in which to set-up a SDM system as shown in Figure 3 that allows for a transformation of the standardized application of EBM (Figure 2) into a dynamic and personalized care pathway.

Structuring the pathway on specific moments of shared decision making—to be applied not only between patients and healthcare professionals, but also between healthcare professionals themselves; our model blends personalized medicine and patient centered care determinants, such as each patients’ genetic features (i.e., the genomic profile), impacts on the patient’s health status (including patient safety and treatment adverse effects) and patient reported measures (PROMs and PREMs).

Additionally, however, following the direction taken by Value-Based Healthcare and, in particular Bodenheimer’s Quadruple-Aim framework [59], the next generation of care pathway’s framework must include also other three dimensions: at an organizational micro level, the framework considers care-team sharing, being a determinant of team wellness; at a macro-organizational level, the principles of green oncology, waste containment and impacts on overall budget and accountability, to evaluate the broadest impact possible of the dynamic pathway.

## 5. Limitations

There are some limitations in this systematic review that should be taken into account when interpreting the results. First of all, broadly speaking, evidence in the current literature of primary studies clearly defining the combined use of personalized medicine and patient-centered care approaches were quite scarce. Secondly, heterogeneity in study designs, in outcome definition and in staging systems adopted (often not at all present in the included studies) prevented us from conducting a meta-analysis and issuing more conclusive results. Furthermore, according to the adopted quality assessment tools, most of the included studies were of moderate–poor quality.

## 6. Conclusions

In the last few years, patient-centered care has been found to be one of the key elements for improving the quality of breast cancer management. Clinical-care-pathways, especially oncological ones, have certainly contributed to the transparency of the decision-making process. The pressure that personalized medicine is placing in the field of oncology, however, highlights the need to structure organizational models that combine PC care models and PM together. The sustainability of the model proposed in this work would largely benefit from further developments and confirmations through additional research, as well as in ulterior organizational contexts.

## Figures and Tables

**Table 1:** Summary results of quality assessment.

Study	Overall %	Quality
Biganzoli et al. 2017 [27]	83%	Group 1
Fountzilas et al. 2018 [25]	83%	Group 1
Rosa 2015 [24]	83%	Group 1
Jacobs et al. 2017 [40]	90%	Group 1
Weldon et al. 2012 [34]	90%	Group 1
Zardavas et al. 2013 [41]	83%	Group 1
Cowppli-Bony et al. 2019 [42]	60%	Group 2
Powis et al. 2017 [44]	60%	Group 2
Wallerstedt et al. 2020 [45]	55%	Group 2
Saini et al. 2019 [31]	67%	Group 2
Schnapper et al. 2018 [37]	70%	Group 2
Komatsu et al. 2014 [39]	70%	Group 2
McGowan et al. 2016 [33]	60%	Group 2
Wright et al. 2019 [35]	60%	Group 2
Kurian et al. 2017 [38]	60%	Group 2
Kurian et al. 2015 [43]	67%	Group 2
Trivedi et al. 2019 [26]	58%	Group 2
Tischler et al. 2019 [29]	42%	Group 3
Girotra et al. 2016 [30]	42%	Group 3
Lyman et al. 2013 [28]	33%	Group 3
Laronga et al. 2012 [32]	50%	Group 3
Roberts et al. 2016 [36]	30%	Group 3
van Hoeve et al. 2014 [48]	27%	Group 3
Al-Naqqash 2020 [46]	45%	Group 3
Perez 2011 [47]	33%	Group 3

**Table 2:** Summary of 25 studies on Breast Cancer management, evaluated in terms of Patient-Centered and Personalized Medicine approaches, assessing success factors and/or barriers. Articles are listed in study design order.

Study	Country	Study Design	Sample (Ethnicity/Age)	Type of BC	PM or/and PC	Dimensions Involved	Barriers	Success Factors
Van Hoeve et al. 2014	The Netherlands	Cohort	NA	Primary BC	Both	Evidence based genetic testing (Shared) clinical decision-making Audit/feedback Evidence based application	Lack of clinicians’ involvement in critical pathways’ construction	Data-derived benchmarking for quality improvements
Cowppli-Bony et al. 2019	France	Cross-sectional	Median age 61	Primary invasive non-metastatic BC	PC	Audit/feedback Evidence based application	Heterogeneous adherence to guidelines among facilities	Data-derived benchmarking for quality improvements
Al-Naqqash 2020	Iraq	Prospective cohort study	Mean age 54	Grade I or grade II cancer, and HER2 negative status	PM	Evidence based genetic testing (Shared) clinical decision-making	Lack of public reimbursement scheme for diagnostics in study context Scarce adherence to guidelines	
Powis et al. 2017	Canada	Retrospective analysis	Age ≥ 65 years	Early-stage BC	PC	Evidence based application	Lack of standardization in treatment and clinical practice	Data-derived benchmarking for quality improvements
McGowan et al. 2016	USA	Qualitative study	Median age 54	Advanced BC	PM	Molecular board	Insufficient integration and engagement of geneticist in MGTB and in counselling Lack of geneticist-patient relationship	Multi-professional and multi-specialist collaboration
Weldon et al. 2012	USA	Qualitative study	NA	Any	PM	Evidence based genetic testing (Shared) clinical decision-making	Lack of public reimbursement scheme for diagnostics in study context Poor timing and seguencing of test relative to decisions Counterincentives to appropriate use of genetic test	Stakeholders’ collaboration with a larger role for patient advocates
Wright et al. 2019	UK	Qualitative study	NA	Any	PM	Molecular board Evidence based genetic testing (Shared) clinical decision-making		Clear allocation of tasks between oncologists and geneticists
Roberts et al. 2016	North Carolina	Qualitative study	NA	Early stage, hormone receptor positive BC, with lymph node negative or lymph node positive disease	PC	Communication	Patients excluded from choices regarding genetic tests	Communication with patients tailored to their needs and background
Schnapper et al. 2018	Italy	Qualitative study	NA	Any	PC	Informative systems	Non-uniform professional profile of DM at EU level	
Kurian et al. 2017	USA	Qualitative study	Black, Asian, Hispanic and white women age 20 to 79 years	Early-stage BC	Both	Evidence based genetic testing Communication (Shared) clinical decision-making	Insufficient integration and engagement of geneticist in counseling Cost barriers to effective testing despite price reduction	Communication with patients tailored to their needs and background
Komatsu et al. 2014	Japan	Qualitative study	NA	Any	Both	Evidence based genetic testing Communication Informative system Multidisciplinary approach	Delay in adoption of new technologies in clinical practice Fragmented communication of genetic data among multidisciplinary professionals	Education and training on genetic testing also among non-geneticists
Jacobs 2017	UK	Delphi study	Median age 53	Any	Both	Molecular board Communication Multidisciplinary approach		Protocols for doctor-patient communication on (pre and post) genetic testing
Kurian 2015		Invited Commentary	NA	Any	PM	Evidence based genetic testing (Shared) clinical decision-making		Shared decision-making between patients and clinicians
Rosa 2015	USA	Narrative review	NA	Any	PM	Molecular board Evidence based genetic testing (Shared) clinical decision-making		Patologists’ knowledge on genetic test
Fountzilas et al. 2018		Narrative review	NA	Any	Both	Evidence based genetic testing Multidisciplinary approach		Education and training on genetic testing
Trivedi et al. 2019		Narrative review	NA	Any	Both	Molecular board Evidence based genetic testing Multidisciplinary approach		Multiprofessional and multispecialist collaboration
Biganzoli et al. 2017		Narrative review	NA	Any	PC	Audit/feedback	Lack of outcome indicators	
Lyman et al. 2013		Narrative review	NA	Any	PM	Molecular board		Multiprofessional and multispecialist collaboration
Tischler et al. 2019		Narrative review	NA	Any	Both	Evidence based genetic testing (Shared) clinical decision-making Communication Multidisciplinary approach	Scarse diffusion of unique vocabulary to guide therapy strategies	Utilization of personalized medicine prevention techniques
Girotra et al. 2016		Narrative review	NA	Any	PM	Evidence based genetic testing (Shared) clinical decision-making		Utilization of personalized medicine prevention techniques
Geetanjali et al. 2019		Narrative review	NA	Any	Both	Evidence based genetic testing (Shared) clinical decision-making Multidisciplinary approach	Dynamicity of genomic data generation and gathering	Multiprofessional and multispecialist collaboration
Laronga et al. 2012		Narrative review	NA	Newly diagnosed, estrogen receptor (ER)-positive, node-negative, early-stage BC treated with endocrine therapy	PM	Molecular board Evidence based genetic testing (Shared) clinical decision-making		Multiprofessional and multispecialist collaboration
Zardavas 2013		Review	NA	Any	PM	Evidence based genetic testing (Shared) clinical decision-making	Lack of systematic approach to the adoption of new technologies in clinical practice	Creation of unique vocabulary to guide therapy strategies
Perez 2011	USA	Review	NA	Any	Both	Molecular board Evidence based genetic testing Multidisciplinary approach	Evidence based on evaluation of personalized medicine only for primary breast cancer	Multiprofessional and multispecialist collaboration
Wallerstedt et al. 2020	Sweden	Systematic review	NA	Post-surgical BC	PM	Evidence based genetic testing (Shared) clinical decision-making	Lack of evidence of therapy effects on HRQL	Utilization of personalized medicine prevention techniques

Legend: NA, not applicable; BC, breast cancer; PM, personalized medicine; PC, patient centered; Early BC, stage 0- I-II. Country is not specified for Invited Commentary, Narrative review and one review.

## Data Availability

Not applicable.

## References

[1] Sung H., Ferlay J., Siegel R.L., Laversanne M., Soerjomataram I., Jemal A., Bray F. (2021). Global cancer statistics 2020: GLOBOCAN estimates of incidence and mortality worldwide for 36 cancers in 185 countries. CA Cancer J. Clin..

[2] Shao J., Rodrigues M., Corter A.L., Baxter N.N. (2019). Multidisciplinary Care of Breast Cancer Patients: A Scoping Review of Multidisciplinary Styles, Processes, and Outcomes. Curr. Oncol..

[3] Cardoso F., Cataliotti L., Costa A., Knox S., Marotti L., Rutgers E., Beishon M. (2017). European Breast Cancer Conference manifesto on breast centres/units. Eur. J. Cancer.

[4] Franceschini G., Di Leone A., Masetti R. (2014). The Breast Unit Update on advantages and the open issues. Ann. Ital. Chir..

[5] Chan C.W.H., Law B.M.H., So W.K.W., Chow K.M., Waye M.M.Y. (2017). Novel Strategies on Personalized Medicine for Breast Cancer Treatment: An Update. Int. J. Mol. Sci..

[6] Cardoso F., Senkus E., Costa A., Papadopoulos E., Aapro M., Andre F., Harbeck N., Lopez B.A., Barrios C., Bergh J. (2018). 4th ESO–ESMO International Consensus Guidelines for Advanced Breast Cancer (ABC 4). Ann. Oncol..

[7] Page M.J., McKenzie J., Bossuyt P., Boutron I., Hoffmann T., Mulrow C., Shamseer L., Tetzlaff J., Akl E., Brennan S. (2020). The PRISMA 2020 Statement: An Updated Guideline for Reporting Systematic Reviews. https://osf.io/preprints/metaarxiv/v7gm2/.

[8] EUR-Lex C:2015:421:FULL. https://eur-lex.europa.eu/legal-content/EN/TXT/?uri=OJ%3AC%3A2015%3A421%3AFULL.

[9] (2001). Medicine I of Crossing the Quality Chasm: A New Health System for the 21st Century. https://www.nap.edu/catalog/10027/crossing-the-quality-chasm-a-new-health-system-for-the.

[10] Naughton C.A. (2018). Patient-Centered Communication. Pharmacy.

[11] Rotter T., Kinsman L., James E.L., Machotta A., Gothe H., Willis J., Snow P., Kugler J. (2010). Clinical pathways: Effects on professional practice, patient outcomes, length of stay and hospital costs. Cochrane Database Syst. Rev..

[12] Yazdanian A., Ayatollahi H., Nahvijou A. (2019). Oncology Information System: A Qualitative Study of Users’ Requirements. Asian Pac. J. Cancer Prev..

[13] Melnyk B.M., Fineout-Overholt E. (2011). Evidence-Based Practice in Nursing & Healthcare: A Guide to Best Practice.

[14] Horvath L.E., Yordan E., Malhotra D., Leyva I., Bortel K., Schalk D., Mellinger P., Huml M., Kesslering C., Huml J. (2010). Multidisciplinary Care in the Oncology Setting: Historical Perspective and Data from Lung and Gynecology Multidisciplinary Clinics. J. Oncol. Pract..

[15] Koopman B., Groen H.J., Ligtenberg M.J., Grünberg K., Monkhorst K., De Langen A.J., Boelens M.C., Paats M.S., Von Der Thüsen J.H., Dinjens W.N. (2020). Multicenter Comparison of Molecular Tumor Boards in The Netherlands: Definition, Composition, Methods, and Targeted Therapy Recommendations. Oncologist.

[16] Pagon R.A., Hanson N.B., Neufeld-Kaiser W., Covington M.L. (2001). Genetic testing. West. J. Med..

[17] Shared Decision Making. https://www.healthit.gov/sites/default/files/nlc_shared_decision_making_fact_sheet.pdf.

[18] Baethge C., Goldbeck-Wood S., Mertens S. (2019). SANRA—A scale for the quality assessment of narrative review articles. Res. Integr. Peer Rev..

[19] Long H.A., French D.P., Brooks J.M. (2020). Optimising the value of the critical appraisal skills programme (CASP) tool for quality appraisal in qualitative evidence synthesis. Res. Methods Med. Health Sci..

[20] Martin J. (2017). Joanna Briggs Institute 2017 Critical Appraisal Checklist for Cohort Studies.

[21] Martin J. (2017). Joanna Briggs Institute 2017 Critical Appraisal Checklist for Text and Opinion.

[22] Stang A. (2010). Critical evaluation of the Newcastle-Ottawa scale for the assessment of the quality of nonrandomized studies in meta-analyses. Eur. J. Epidemiol..

[23] Martin J. (2017). Joanna Briggs Institute 2017 Critical Appraisal Checklist for Systematic Reviews and Research Syntheses.

[24] Rosa M. (2015). Advances in the Molecular Analysis of Breast Cancer: Pathway toward Personalized Medicine. Cancer Control.

[25] Fountzilas C., Kaklamani V.G. (2018). Multi-gene Panel Testing in Breast Cancer Management. Cancer Treat. Res..

[26] Trivedi H., Kling H.M., Treece T., Audeh W., Srkalovic G. (2019). Changing Landscape of Clinical-Genomic Oncology Practice. Acta Medica Acad..

[27] Biganzoli L., Marotti L., Hart C.D., Cataliotti L., Cutuli B., Kühn T., Mansel R.E., Ponti A., Poortmans P., Regitnig P. (2017). Quality indicators in breast cancer care: An update from the EUSOMA working group. Eur. J. Cancer.

[28] Lyman G.H., Baker J., Geradts J., Horton J., Kimmick G., Peppercorn J., Pruitt S., Scheri R.P., Hwang E.S. (2013). Multidisciplinary Care of Patients with Early-Stage Breast Cancer. Surg. Oncol. Clin. N. Am..

[29] Tischler J., Crew K.D., Chung W.K. (2019). Cases in Precision Medicine: The Role of Tumor and Germline Genetic Testing in Breast Cancer Management. Ann. Intern. Med..

[30] Girotra S., Yeghiazaryan K., Golubnitschaja O. (2016). Potential biomarker panels in overall breast cancer management: Advancements by multilevel diagnostics. Pers. Med..

[31] Saini G., Mittal K., Rida P., Janssen E.A.M., Gogineni K., Aneja R. (2019). Panoptic View of Prognostic Models for Personalized Breast Cancer Management. Cancers.

[32] Laronga C., Harness J.K., Dixon M., Borgen P.I. (2012). The role of the breast cancer surgeon in personalized cancer care: Clinical utility of the 21-gene assay. Am. J. Surg..

[33] McGowan M.L., Ponsaran R.S., Silverman P., Harris L.N., Marshall P.A. (2016). “A rising tide lifts all boats”: Establishing a multidisciplinary genomic tumor board for breast cancer patients with advanced disease. BMC Med Genom..

[34] Weldon C.B., Trosman J.R., Gradishar W.J., Benson A.B., Schink J.C. (2012). Barriers to the Use of Personalized Medicine in Breast Cancer. J. Oncol. Pract..

[35] Wright S., Porteous M., Stirling D., Young O., Gourley C., Hallowell N. (2019). Negotiating jurisdictional boundaries in response to new genetic possibilities in breast cancer care: The creation of an ‘oncogenetic taskscape’. Soc. Sci. Med..

[36] Roberts M.C., Bryson A., Weinberger M., Dusetzina S., Dinan M.A., Reeder-Hayes K.E., Wheeler S.B. (2016). Patient-Centered Communication for Discussing Oncotype DX Testing. Cancer Investig..

[37] Schnapper G., Marotti L., Casella D., Mano M.P., Mansel R.E., Ponti A., The EUSOMA Breast Centers Network Data Managers (2018). Data managers: A survey of the European Society of Breast Cancer Specialists in certified multi-disciplinary breast centers. Breast J..

[38] Kurian A.W., Li Y., Hamilton A.S., Ward K.C., Hawley S.T., Morrow M., McLeod M.C., Jagsi R., Katz S.J. (2017). Gaps in Incorporating Germline Genetic Testing into Treatment Decision-Making for Early-Stage Breast Cancer. J. Clin. Oncol..

[39] Komatsu H., Yagasaki K. (2013). Are we ready for personalized cancer risk management? The view from breast-care providers. Int. J. Nurs. Pract..

[40] Jacobs C., Pichert G., Harris J., Tucker K., Michie S. (2017). Key messages for communicating information about BRCA1 and BRCA2 to women with breast or ovarian cancer: Consensus across health professionals and service users. Psycho-Oncology.

[41] Zardavas D., Pugliano L., Piccart M. (2013). Personalized therapy for breast cancer: A dream or a reality?. Futur. Oncol..

[42] Cowppli-Bony A., Trétarre B., Marrer E., Defossez G., Daubisse-Marliac L., Coureau G., Minicozzi P., Woronoff A.-S., Delafosse P., Molinié F. (2019). Compliance with clinical guidelines for breast cancer management: A population-based study of quality-of-care indicators in France. PLoS ONE.

[43] Kurian A.W., Friese C.R. (2015). Precision Medicine in Breast Cancer Care: An Early Glimpse of Impact. JAMA Oncol..

[44] Powis M., Sutradhar R., Gonzalez A., Enright K.A., Taback N.A., Booth C.M., Trudeau M., Krzyzanowska M.K. (2017). Establishing achievable benchmarks for quality improvement in systemic therapy for early-stage breast cancer. Cancer.

[45] Wallerstedt S.M., Ek A.N., Bagge R.O., Kovács A., Strandell A., Linderholm B. (2020). Personalised medicine and the decision to withhold chemotherapy in early breast cancer with intermediate risk of recurrence—A systematic review and meta-analysis. Eur. J. Clin. Pharmacol..

[46] Al-Naqqash M.A. (2020). The 21-gene oncotype DX offers more accurate treatment decisions in early breast cancer. Gastric Breast Cancer.

[47] Perez E.A. (2011). Breast Cancer Management: Opportunities and Barriers to an Individualized Approach. Oncologist.

[48] Van Hoeve J., De Munck L., Otter R., De Vries J., Siesling S. (2014). Quality improvement by implementing an integrated oncological care pathway for breast cancer patients. Breast.

[49] El-Alti L., Sandman L., Munthe C. (2017). Person Centered Care and Personalized Medicine: Irreconcilable Opposites or Potential Companions?. Health Care Anal..

[50] Nardini C., Osmani V., Cormio P.G., Frosini A., Turrini M., Lionis C., Neumuth T., Ballensiefen W., Borgonovi E., D’Errico G. (2021). The evolution of personalized healthcare and the pivotal role of European regions in its implementation. Pers. Med..

[51] Maes-Carballo M., Martín-Díaz M., Mignini L., Khan K., Trigueros R., Bueno-Cavanillas A. (2021). Evaluation of the Use of Shared Decision Making in Breast Cancer: International Survey. Int. J. Environ. Res. Public Health.

[52] Panella M., Marchisio S., Di Stanislao F. (2003). Reducing clinical variations with clinical pathways: Do pathways work?. Int. J. Qual. Health Care.

[53] Smeds M. (2019). Managing Care Pathways for Patients with Complex Care Needs.

[54] Elwyn G., Laitner S., Coulter A., Walker E., Watson P., Thomson R. (2010). Implementing shared decision making in the NHS. BMJ.

[55] Levit L., Balogh E., Nass S., Ganz P.A., Committee on Improving the Quality of Cancer Care: Addressing the Challenges of an Aging Population, Board on Health Care Services, Institute of Medicine (2013). Delivering High-Quality Cancer Care: Charting a New Course for a System in Crisis.

[56] Elwyn G., Frosch D., Thomson R., Joseph-Williams N., Lloyd A., Kinnersley P., Cording E., Tomson D., Dodd C., Rollnick S. (2012). Shared Decision Making: A Model for Clinical Practice. J. Gen. Intern. Med..

[57] Schoenfeld E.M., Mader S., Houghton C., Wenger R., Probst M.A., Schoenfeld D.A., Lindenauer P.K., Mazor K.M. (2019). The Effect of Shared Decisionmaking on Patients’ Likelihood of Filing a Complaint or Lawsuit: A Simulation Study. Ann. Emerg. Med..

[58] Maes-Carballo M., Muñoz-Núñez I., Martín-Díaz M., Mignini L., Bueno-Cavanillas A., Khan K.S. (2020). Shared decision making in breast cancer treatment guidelines: Development of a quality assessment tool and a systematic review. Health Expect..

[59] Bodenheimer T., Sinsky C. (2014). From Triple to Quadruple Aim: Care of the Patient Requires Care of the Provider. Ann. Fam. Med..

